# Effects of Water Cup Cleaning on Drinking Behaviour, Water Intake, and Milk Yield of Lactating Dairy Cows in Tie-Stall Barns

**DOI:** 10.3390/ani16111634

**Published:** 2026-05-27

**Authors:** Yurina Yamane, Natsuki Yoshioka, Tetsuya Seo

**Affiliations:** Department of Animal Science, Obihiro University of Agriculture and Veterinary Medicine, Obihiro 080-8555, Japan; yamane@obihiro.ac.jp (Y.Y.);

**Keywords:** drinking behavior, drinking efficiency, water intake, water hygiene

## Abstract

Water cups in tie-stall barns often remain uncleaned for long periods, allowing organic matter and biofilms to accumulate, which may reduce drinking efficiency, defined as water intake per drinking event rather than drinking frequency, and increase hygienic risks, including potential bacterial contamination, in dairy cows. This study examined the short-term effects of cleaning heavily fouled water cups on drinking behaviour, water intake, and milk yield in lactating Holstein cows. Twenty-four cows were observed on a baseline day and again after a one-day adaptation period following cup cleaning, using a within-animal comparison. Cleaning significantly increased total daily water intake by approximately 5 L (*p* < 0.05) and water intake per drinking bout by approximately 2 L (*p* < 0.01), while drinking frequency, milk yield, and maintenance behaviours remained unchanged. Temperature–humidity conditions were similar across observation days, indicating that increased intake was attributable to improved water hygiene rather than environmental variation. Although cleaning enhanced drinking efficiency, the magnitude of increased water intake was insufficient to affect short-term milk yield. These findings demonstrate that regular water cup cleaning is a practical management practice that improves drinking efficiency and water hygiene, with potential implications for cow health and welfare.

## Simple Summary

Dairy cows kept in tie-stall barns usually drink from small water cups that release water when the cow pushes a valve. If these cups are not cleaned regularly, feed particles, slime, and algae can build up. Dirty cups may make cows less willing to drink or cause them to drink less efficiently. In this study, we cleaned heavily fouled water cups and observed how cows changed their drinking behaviour. Each cow was compared with herself before and after cleaning. After the cups were scrubbed, cows drank more water each time they visited the cup, which increased their total daily water intake. However, milk yield did not differ before and after cleaning during the short-term observation period. These results indicate that cleaning water cups can improve drinking efficiency and water hygiene even in the absence of short-term changes in milk yield. Regular cleaning of water cups is therefore a practical and useful management practice that supports appropriate daily management and animal welfare in tie-stall systems.

## 1. Introduction

Water is a fundamental nutrient for animals, including cattle. Experimental restriction of water availability alters physiological responses in growing and fattening cattle, resulting in reduced body weight and feed intake [1]. Restricted water intake has also been shown to reduce feed consumption and impair nutrient utilization in Zebu cattle [2]. In lactating cows, limiting water availability to 50% of the normal intake has been shown to reduce lying time and milk yield [3]. A recent review emphasized that insufficient water access or poor water quality can reduce milk production and compromise animal welfare [4].

Water intake in dairy cows is influenced by multiple nutritional and environmental factors. According to NASEM [5], water requirements depend primarily on dry matter intake, milk yield, diet composition, and ambient temperature. Previous studies indicate that water intake in lactating Holstein cows averages around 80 L/day and may reach approximately 100 L/day depending on production level and environmental conditions [6,7].

Season has also been reported to influence drinking behaviour. Holter and Urban [8] observed a seasonal trend in free water intake, with higher values in June and lower values in December. Similarly, Moncada and Hsia [9] reported average intakes of approximately 62 L/day in summer and 39 L/day in winter under tropical conditions. Although these values are lower than those reported for high-producing cows fed drier TMR, they provide useful reference information on seasonal variation in water intake.

In tie-stall barns, dairy cows typically drink from push-valve water cups, often with a cup positioned between two adjacent cows. Poor water quality in outdoor sources can reduce intake and performance because cattle tend to avoid contaminated dugouts and prefer clean trough water [10]. On many commercial farms, water cups remain uncleaned for prolonged periods, allowing organic matter and biofilms to accumulate and creating niches for pathogens [11,12]. Continued use of contaminated cups may suppress cows’ drinking behaviour, and regular scrubbing and proper management are necessary to ensure water quality. Japanese national guidelines emphasize the importance of hygienic management of watering facilities [13], although they do not provide objective criteria for assessing water cup cleanliness in tie-stall barns. Practical criteria such as regular cleaning frequency and visible assessment of contamination are required in farm management.

Recent research indicates that drinking facilities on dairy farms can become microbially contaminated, particularly when biofilm, an adhesive matrix formed by microorganisms attached to surfaces, accumulates on trough surfaces. High temperatures are associated with increased bacterial contamination, which may allow dirty troughs to act as potential reservoirs of pathogenic and antimicrobial-resistant bacteria [14]. Such contamination may reduce water palatability and discourage drinking, as cows may avoid water with unpleasant odour or taste, and may also increase the risk of waterborne infections. This evidence suggests a dual impact of poor water hygiene on both drinking behaviour and animal health.

In loose housing systems, cows move freely and waterers are more exposed to manure contamination, which increases the risk of waterborne infections. In contrast, cows in tie-stall barns are individually restrained and have access to only a single water cup, meaning they cannot avoid contaminated drinking devices. Cleaning of drinking devices has been shown to alter drinking behaviour in free-stall and pasture systems [15,16], however, these studies did not assess milk yield. Furthermore, none have evaluated the effects of water cup cleaning on drinking behaviour or milk yield in tie-stall barns without access to alternative water sources. This represents an important gap in understanding how water cup hygiene affects hydration and productivity under confined housing conditions.

We therefore hypothesized that cleaning heavily fouled water cups would improve drinking efficiency and increase water intake in lactating dairy cows kept in tie-stall barns. To test this hypothesis, we conducted a within-animal comparison under similar environmental conditions, measured drinking behaviour, water intake, and milk yield before and after cleaning. This framework was designed to isolate the immediate effects of improved water hygiene on drinking behaviour and water intake dynamics in dairy cows.

## 2. Materials and Methods

### 2.1. Study Farm

The study was conducted on a commercial dairy farm located in the Tokachi region of Hokkaido, Japan. The herd consisted of 73 Holstein cows housed in a tie-stall barn with a tie-rail system (commonly referred to as a New York–style tie stall). The average annual milk yield was 11,402 kg per cow, with two cows sharing each water cup. Feed was provided by an automatic feeder seven times daily, at 04:00–05:00, 06:20–07:20, 09:30–10:30, 12:30–13:30, 15:30–16:30, 19:00–20:00, and 22:00–23:00.

Cows were fed a total mixed ration (TMR) consisting of 40–50 kg of corn silage and grass silage mixed at a 3:2 ratio (weight/weight), supplemented with 3 kg of beet pulp, concentrate, and rumen-undegradable protein (RUP). Concentrate was initially offered at 4 kg immediately after calving and increased by 0.25 kg per day until peak lactation. At peak lactation, concentrate allowance was 9.5 kg for primiparous cows, 10.5 kg for second-lactation cows, and 11.0 kg for cows in their third or later lactations. RUP was supplied at 1.0 kg for primiparous cows, 1.5 kg for second-lactation cows, and 2.0 kg for cows in their third or later lactations, and was discontinued once the cows were confirmed pregnant. All feed amounts are expressed on an as-fed basis, per cow per day. Cows were milked twice daily, at 05:20–06:50 and 16:45–18:15, using a pipeline milking system. Teats were cleaned and dried before milking, and post-milking teat disinfection was performed after cluster removal.

### 2.2. Study Design

The study was conducted over two separate periods totaling 48 days, from 5 to 31 October and from 5 to 25 November 2019. Each cow was subjected to a three-day observation period. On Day 1, water intake, milk yield, and behaviour were recorded using water cups that had not been cleaned. Prior to this observation, the inner surfaces of the water cups at the study farm had not been scrubbed for several years; thus, the Day 1 measurements reflected a long-term unclean condition. Subsequently, baking soda was sprinkled inside the water cups, and they were scrubbed with a brush to ensure cleanliness. After cleaning, all water cups were thoroughly rinsed with clean water to remove any residual baking soda and to minimize potential effects on taste or odour. Day 2 served as an adaptation period to the cleaned water cups, during which no measurements were taken. On Day 3, water intake, milk yield, and behaviour were recorded again using the cleaned water cups. This protocol enabled within-animal comparisons between pre- and post-cleaning conditions.

### 2.3. Water-Cup Fouling Score

Water cup cleanliness was assessed based on the Animal Welfare Assessment Protocol for Dairy Cattle [17], which defines drinking facilities as being free from contamination by spoiled feed particles, slime, feces, algae, or similar substances. Four contamination types—spoiled feed particles, slime inside the cup, slime on the back of the pusher plate, and algae growth—were evaluated. Each contamination type was scored on an ordinal scale from 0 (no contamination) to 2 or 3, depending on the contamination category defined in Table 1, with higher scores indicating more severe fouling. The fouling score for each water cup was calculated as the sum of the scores for the four contamination types.

### 2.4. Experimental Cows

A total of 38 iron push-valve–type water cups were installed in the tie-stall barn. Of these, 6 cups had a capacity of 4600 mL, while the remaining 32 had a capacity of 4000 mL. This study focused exclusively on the 32 cups of identical capacity. Immediately before the start of the survey on 1 October 2019, these cups were scored according to the fouling categories defined in Table 1 to determine the fouling score of each cup.

Lactating cows using the selected cups were excluded if they met any of the following criteria:

a. Insufficient milk records or cows not past their estimated peak lactation day: fewer than three official test-day milk records, or not yet past their estimated peak lactation day, as determined by the Multiple Trait Prediction (MTP) method based on Wilmink’s lactation curve model [18]. These criteria were applied because reliable estimation of lactation performance requires multiple records, and cows in early lactation experience increasing milk yield that strongly drives water intake [19], making it difficult to isolate the effect of water cup cleaning on production.
b. Mastitis treatment: cows under treatment for mastitis, as the disease induces a decline in milk yield and would confound evaluation of the effect of water cup cleaning [20].
c. Advanced lactation: cows at ≥300 days in milk, as they were likely to enter the dry period and leave the barn during the study.

After applying these criteria, 28 lactating cows using 16 water cups with fouling scores of 3–5 were retained in the final experimental cohort. The mean fouling score of the selected water cups was 4.4 ± 0.6 (mean ± SD).

### 2.5. Water Intake Measurement

Water intake was recorded on the observation days described in Section 2.2 during four daily intervals—04:00–08:00, 09:30–11:30, 12:30–14:30, and 15:30–18:30—for a total of 11 h per day. Because drinking frequency and volume increase in conjunction with feeding [21], and peak intake coincides with both feeding and milking times [6,22,23,24], each recording period was aligned with these events.

Data collection began when the automatic feeder started operating. For the first three intervals, recording ceased one hour after the final feeding; for the 15:30–18:30 interval, it ended 15 min after the last milking. Milking occurred during both the 04:00–08:00 and 15:30–18:30 periods. The detailed procedures for measuring water intake are described in the following sections.

#### 2.5.1. Water Intake per Drinking Event

Water intake per bout was calculated as:

residual volume before drinking + dispensed volume − residual volume after drinking, where dispensed volume was determined by multiplying the cup’s mean flow rate (L/s) by the duration of water flow (s).

#### 2.5.2. Residual Water Volume in the Water Cup

A study was conducted to derive an equation for estimating residual water volume from the water level in the cup. Ten cups were randomly selected from the 32 in use. Starting from an empty state, water was added in 100 mL increments, and the water level (cm) was measured with a ruler after each addition until the cup was full.

Based on the recorded water-level and volume data, the following regression equation was developed:

Residual water volume (L) = 0.0287 × (water level in cm)^2^ + 0.1146 × (water level in cm) (R^2^ = 0.99).

#### 2.5.3. Flow Rate of Water Cups

The mean flow rates of all 16 cups used by the experimental cows were determined. The day before the main observation, a preliminary test was conducted to establish the average flow rate for each of the four time periods.

For each cup, the flow rate was measured three times per period. Specifically, each cup was filled to capacity, and the paddle was pressed for 10 s; the overflow was collected in a bucket and weighed to record the dispensed volume (L/10 s). The recorded volumes were then converted to flow rates in L/s. For each cup, the interval-specific mean flow rates (L/s) were applied in the water intake calculations.

The flow rates ranged from 0.19 to 0.33 L/s (11.4–19.8 L/min), with all cups meeting the minimum requirement of 10 L/min for drinking facilities specified by Welfare Quality^®^ [25], Zappavigna et al. [26], and RSPCA [27], which apply broadly to drinking facilities.

Using the above method, first the residual water level in the cup was measured with a ruler before drinking behaviour, then the duration of the drinking event was timed when drinking was observed, and finally the residual water level was measured again after drinking to calculate the intake for each drinking event.

### 2.6. Behavioural Observation

Prior to data collection, both observers were trained together at the study farm to ensure consistency in their assessments. Behavioural observations were primarily conducted by a single trained observer, and a second observer provided supplementary coverage during short breaks to avoid fatigue. Although formal inter-rater reliability testing was not conducted, the observers were trained together and periodically cross-checked their observations to ensure consistency.

Observations were conducted from approximately 1.5 m in front of each stall, allowing a clear view of the cow’s muzzle and the water cup without disturbing the animals. The barn was illuminated by natural daylight from side windows, supplemented by ceiling-mounted LED lighting to maintain consistent visibility throughout all observation periods.

Behavioural observations were conducted concurrently with water-intake measurements during the same four daily intervals described above (totaling 11 h per day). Each observation day, one or two adjacent cows sharing the same water cup were observed. Drinking behaviour was recorded only when the experimenter was not performing milk-yield measurements, and was defined as starting when the cow’s muzzle contacted the water surface and ending when it was lifted away. Water-flow duration (timed with a stopwatch) and post-drinking water level (measured with a ruler) were recorded for each drinking event, except when the duration was too brief to confirm drinking. A predefined ethogram was used to classify non-drinking behaviours during the observation periods. Feeding was defined as head inside the feed trough while ingesting or manipulating feed. Rumination was defined as rhythmic chewing of cud and was recorded separately in standing and lying positions. Resting was defined as inactive behaviour without feeding or ruminating and was also distinguished between standing and lying postures. Standing was defined as upright with all four hooves bearing weight, and lying was defined as the abdomen or thorax in contact with the floor. These behaviours and postures were recorded by direct visual observation at 5-min intervals using a time-sampling method.

### 2.7. Milk Yield Measurement

Milk yield was measured on the same days as water intake measurements. At each morning and evening milking, a milk meter (Milk Meter FV; TRU-TEST, Auckland, New Zealand) was attached to the milking unit, and the yield at each milking was recorded to calculate daily milk yield.

### 2.8. Cleaning of the Water Cups

After completing the measurements on Day 1, the water cups were cleaned. Baking soda was sprinkled into each cup, and they were scrubbed with a brush [28]. Cleaning continued until all four contamination scores shown in Table 1 were zero.

### 2.9. Temperature and Humidity

Ambient temperature and relative humidity inside the barn were measured using an environmental meter (LM-8010; FUSO Co., Ltd., Tokyo, Japan). Measurements were recorded at 30-min intervals from the start of behavioural observations until the end of the study, and the mean of these values was calculated. The temperature–humidity index (THI) was then calculated as follows:

THI = 0.8 × T + 0.01 × RH × (T − 14.3) + 46.3,

where T is ambient temperature (°C) and RH is relative humidity (%). This formula was selected because it is widely used in cattle heat-stress research and is suitable for evaluating thermal conditions in humid barn environments [29].

### 2.10. Statistical Analysis

Comparisons of water intake, drinking frequency, behaviours, postures, daily milk yield, and THI before and after cup cleaning were performed using the Wilcoxon signed-rank test because the data consisted of paired measurements from the same cows and did not satisfy the assumptions required for parametric tests. Correlations between THI and daily water intake, and between THI and water intake per bout, were assessed using Spearman’s rank correlation coefficient, as these variables showed non-normal distribution patterns typical of behavioural and intake data. For all analyses, a significance level of *p* < 0.05 was adopted.

Twenty-eight cows using 16 cups were investigated; two were excluded because they exhibited non-consumptive pressing of the drinker without ingesting water [30] or water lapping [31,32,33,34], behaviours that caused excessive overflow from the cups, preventing accurate measurement. Two additional cows were excluded because drinking frequency during milk-yield recording exceeded 20% of total drinking frequency. The final analysis therefore included 24 cows using 15 cups. The mean parity of these cows was 3.5 ± 1.8 (SD), and the mean days in milk (DIM) was 204.7 ± 69.8 (SD). The characteristics of the cows and their milk yield during the observation period are summarized in Table 2.

## 3. Results

### 3.1. Environmental Conditions

In the present study, treatments were conducted on consecutive days under similar ambient and water temperatures. The mean temperature–humidity index (THI; min–max) was 56.2 (41.0–68.6) before cleaning and 55.4 (41.4–66.6) after cleaning, with both values well below 72, a value above which adverse effects of heat stress on high-producing dairy cows have been reported [35]. No significant difference in mean THI was detected between the two treatment days (Wilcoxon signed-rank test, *p* = 0.30).

Spearman’s rank correlation revealed no significant association between mean THI and total water intake (pre-cleaning: r_s_ = 0.00, *p* = 0.98; post-cleaning: r_s_ = −0.21, *p* = 0.30). Similarly, no significant association was observed between mean THI and water intake per drinking bout (pre-cleaning: r_s_ = 0.01, *p* = 0.95; post-cleaning: r_s_ = −0.20, *p* = 0.32).

### 3.2. Drinking Behaviour and Water Intake

Table 3 shows changes in drinking behaviour before and after water cup cleaning. The number of drinking bouts showed no significant difference between treatments. In contrast, total water intake increased significantly after cleaning (*p* < 0.05), with cows consuming approximately 5 L more water per day.

Water intake per drinking bout also increased significantly following water cup cleaning (*p* < 0.01), with cows consuming approximately 2 L more water per bout. Considerable variation among cows was observed for drinking behaviour and water intake variables, as reflected by the relatively large standard deviations (Table 3).

### 3.3. Milk Yield

Daily milk yield did not differ significantly before and after water cup cleaning (*p* > 0.05). Mean milk yield remained approximately 33 kg/day under both conditions (Table 3).

## 4. Discussion

The present study demonstrated that cleaning water cups increased total water intake and water intake per drinking bout in lactating cows housed in a tie-stall barn, while the number of drinking bouts remained unchanged. These findings suggest that water cup cleaning improved drinking efficiency without altering drinking frequency.

Previous studies comparing environmental conditions have shown that drinking water intake in dairy cows varies widely depending on temperature and physiological status. Meyer et al. [36] reported substantial individual variation in daily water intake, ranging from 14 to 171 kg/day, with higher ambient temperatures and higher milk yield significantly increasing water intake. Similarly, Krauß et al. [37] observed large differences in water intake between cows housed in different milking systems. In the present study, temperature–humidity index values remained within a moderate range on both observation days, indicating that climatic conditions were relatively stable. Accordingly, the observed increases in water intake are unlikely to have been driven by environmental variation and are more plausibly attributed to the direct effects of water cup cleaning.

The increase in water intake following cleaning occurred primarily through an increase in water intake per drinking bout rather than an increase in drinking frequency. This finding indicates that water cup cleaning improved drinking efficiency by altering qualitative aspects of drinking behaviour rather than increasing the motivation to drink. This pattern is consistent with previous studies showing that improved water cleanliness affects drinking characteristics such as intake per bout, pauses, and exploratory behaviours, without necessarily increasing drinking frequency [15,16].

The present study extends these findings to a tie-stall barn with individual water cups, demonstrating that improved water hygiene can enhance drinking efficiency even in the absence of alternative water sources or social competition for access.

Large individual variation in drinking behaviour was observed in the present study, as reflected by the wide standard deviations in drinking bout frequency and water intake variables. Such variation may reflect inherent differences among cows in water requirements and drinking patterns. In group-housed dairy cows, high ambient temperatures have been shown to increase competition at drinkers, resulting in more frequent displacements and altered drinking patterns [38]. In such systems, automated longitudinal monitoring has further demonstrated that social hierarchy influences drinking frequency, timing, and daily water intake, with subordinate cows modifying drinking behaviour to avoid peak competition periods [39]. In contrast, cows in the present tie-stall barn did not compete for access to water cups, suggesting that the observed variability was more likely related to intrinsic differences in drinking strategy, metabolic demand, and habitual patterns of water use rather than social factors.

The fouling scores of the water cups ranged from 3 to 5, indicating moderate to high levels of contamination prior to cleaning. However, the extent to which this variability influenced the magnitude of the observed effects was not directly assessed in this study and therefore remains unclear. In general, contamination of drinking facilities may reduce water palatability and consequently influence intake and drinking behaviour. Thus, differences in the degree of contamination may lead to variation in the magnitude of changes in water intake and drinking patterns. Therefore, the magnitude of the effects observed in this study may have been partly influenced by differences in the pre-cleaning contamination status of the water cups.

Despite the observed increase in water intake, daily milk yield did not differ before and after water cup cleaning. This finding should be interpreted in the context of the already high production level of the cows and the moderate climatic conditions during the study period. The cows in the present study produced approximately 33 kg of milk per day on average, which is at the higher end of the range typically reported for commercial Holstein herds in Japan under thermoneutral conditions. Previous studies have shown that responses of milk yield to changes in water-related conditions are most pronounced under conditions of severe water restriction or environmental heat stress, whereas under non-stressful conditions, short-term or moderate changes in water availability may alter drinking behaviour without affecting milk yield [3,24,35,40]. Under the conditions of the present study, therefore, the primary benefits of water cup cleaning are better interpreted as improvements in drinking efficiency and water hygiene rather than immediate increases in milk production.

Several limitations of this study should be considered. The experimental design did not include a negative control (i.e., water cups that were not cleaned) because, after applying the selection criteria described in the Materials and Methods, the number and configuration of eligible cows and water cups did not allow the establishment of an independent control group of sufficient size. Therefore, a within-animal comparison design was adopted to control for individual variability. Consequently, the observed effects may have been influenced not only by the cleaning treatment but also by day-to-day variation. Although the within-animal comparison design reduced individual variability, potential confounding effects associated with the sequential measurement design cannot be fully excluded.

The study was conducted on a single commercial farm with a limited number of cows, and only one type of water cup was evaluated, which may limit the generalizability of the findings. Dry matter intake was not measured, and the experiment was based on single-day observations, making it difficult to account for temporal variation. Future studies should include longer observation periods covering multiple days in order to capture temporal variation and longer-term responses.

Microbiological analyses and comparisons among different cleaning methods were not performed, as this was beyond the scope of the present study and constrained by practical limitations in on-farm sampling. In addition, residual odor after cleaning, particularly due to the use of baking soda, may have influenced drinking behaviour.

The relatively high fouling scores before cleaning (3–5) suggest that the observed response may represent the upper range of the effect. The experiment was conducted under relatively stable autumn climatic conditions, allowing the isolation of short-term effects of water cup cleaning while minimizing confounding influences such as heat stress. However, drinking behaviour and water intake are influenced by heat stress, and therefore the responses observed here may differ under summer conditions. Furthermore, fouling scores were not re-assessed after cleaning due to the short observation period and practical constraints, which limited the evaluation of re-contamination dynamics.

In addition to enhancing drinking efficiency, regular cleaning of water cups may contribute to improved hygiene and a reduced risk of infection associated with contaminated drinking surfaces. These practical benefits may be particularly important under warm conditions, when microbial growth is promoted and adequate water intake becomes critical.

## 5. Conclusions

In lactating dairy cows housed in tie-stall barns, cleaning the water cups increased water intake per bout and total intake, improving drinking efficiency without affecting drinking frequency, behaviour, or milk yield. These effects appear to be directly attributable to improved water hygiene at the drinking point. From a practical perspective, regular cleaning of water cups should be incorporated into routine farm management to support water hygiene and animal welfare. However, further studies are needed to evaluate the long-term impacts of improved water-cup hygiene on cow health, feed intake, and productivity under different management and climatic conditions.

## Figures and Tables

**Table 1:** Evaluation categories and scoring system for water cup fouling.

Score	Contamination Type	Definition
0	Spoiled feed particles	No spoiled feed particles present in the cup
1		A small amount of spoiled feed particles present on the bottom of the cup
2		Spoiled feed particles covering the entire bottom surface of the cup
0	Slime inside cup	No slime present
1		Slime present directly below the spout
2		Slime present in areas other than directly below the spout
0	Slime on back of pusher plate	No slime present.
1		Slime present up to 3 cm from the paddle tip
2		Slime present up to 10 cm from the paddle tip (approximately half the paddle length)
3		Slime covering the entire paddle surface.
0	Algae growth	No algae present
1		Algae present directly below the spout
2		Algae present in areas other than directly below the spout

**Table 2:** Characteristics of the cows and milk yield during the observation period.

Cow ID	Parity	DIM (Days)	Milk Yield (kg/day)		
			Mean ± SD	Minimum	Maximum
690	7	285	32.8 ± 0.7	32.0	33.6
702	6	76	47.0 ± 2.3	44.4	50.0
717	7	217	30.1 ± 1.3	28.4	31.4
726	6	105	30.2 ± 1.2	28.7	31.6
756	6	190	30.5 ± 2.2	27.6	32.9
825	5	283	23.3 ± 0.6	22.8	24.2
880	4	148	45.0 ± 0.7	44.5	46.0
904	4	277	13.0 ± 0.4	12.6	13.5
909	4	207	35.4 ± 1.5	34.1	37.5
953	4	139	45.0 ± 1.5	43.0	46.5
958	3	283	35.7 ± 0.6	34.9	36.4
961	3	255	33.6 ± 0.9	32.5	34.6
963	3	115	46.1 ± 1.8	43.8	48.3
964	3	181	34.7 ± 0.4	34.2	35.0
965	3	208	36.8 ± 0.8	35.7	37.5
1006	3	124	29.2 ± 0.2	29.0	29.4
1007	3	176	41.0 ± 1.4	39.3	42.8
1015	2	157	45.0 ± 0.7	44.1	45.6
1016	2	282	29.0 ± 1.3	27.5	30.6
1018	2	290	27.0 ± 0.0	27.0	27.1
1105	2	125	34.7 ± 0.8	33.9	35.8
1122	1	287	24.8 ± 1.0	23.5	26.0
1123	1	285	20.5 ± 0.4	20.0	21.1
1131	1	218	30.9 ± 0.9	29.7	31.9

**Table 3:** Drinking behaviour, water intake, and milk yield of dairy cows before and after water cup cleaning (mean ± SD).

	Pre-Cleaning	Post-Cleaning	*p*-Value ^2^
Number of drinking bouts	13.0 ± 13.0	12.1 ± 13.0	ns
Total water intake (L/day)	83.1 ± 30.5	88.8 ± 30.6	*
Water intake per drinking bout (L) ^1^	10.2 ± 6.2	12.4 ± 8.4	**
Milk yield (kg/day)	33.5 ± 8.8	33.2 ± 8.4	ns

^1^ Calculated as total water intake (L) divided by the number of drinking bouts. ^2^ Wilcoxon signed-rank test. * *p* < 0.05; ** *p* < 0.01; ns, not significant.

## Data Availability

The data presented in this study are available from the corresponding author upon reasonable request.

## Abbreviations

The following abbreviations are used in this manuscript:

DIM	Days in Milk
DMI	Dry Matter Intake
MTP	Multiple Trait Prediction
RUP	Rumen-Undegradable Protein
THI	Temperature–Humidity Index
TMR	Total Mixed Ration
